# Restart Mechanisms for the Successive-Cancellation List-Flip Decoding of Polar Codes

**DOI:** 10.3390/e27030309

**Published:** 2025-03-14

**Authors:** Charles Pillet, Ilshat Sagitov, Alexios Balatsoukas-Stimming, Pascal Giard

**Affiliations:** 1 LaCIME, Department of Electrical Engineering, École de technologie supérieure (ÉTS), 1100 Notre-Dame St. West, Montréal, QC H3C 1K3, Canada; ilshat.sagitov.1@ens.etsmtl.ca; 2 Department of Electrical Engineering, Eindhoven University of Technology, Groene Loper 19, 5600 MB Eindhoven, The Netherlands; a.k.balatsoukas.stimming@tue.nl

**Keywords:** polar codes, decoding, execution time, complexity, energy efficiency

## Abstract

Polar codes concatenated with a cyclic redundancy check (CRC) code have been selected in the 5G standard with the successive-cancellation list (SCL) of list size L = 8 as the baseline algorithm. Despite providing great error-correction performance, a large list size increases the hardware complexity of the SCL decoder. Alternatively, flip decoding algorithms were proposed to improve the error-correction performance with a low-complexity hardware implementation. The combination of list and flip algorithms, the successive-cancellation list flip (SCLF) and dynamic SCLF (DSCLF) algorithms, provides error-correction performance close to SCL-32 with a list size L = 2 and $T_{\max}$ = 300 maximum additional trials. However, these decoders have a variable execution time, a characteristic that poses a challenge to some practical applications. In this work, we propose a restart mechanism for list–flip algorithms that allows us to skip parts of the decoding computations without affecting the error-correction performance. We show that the restart location cannot realistically be allowed to occur at any location in a codeword as it would lead to an unreasonable memory overhead under DSCLF. Hence, we propose a mechanism where the possible restart locations are limited to a set and propose various construction methods for that set. The construction methods are compared, and the tradeoffs are discussed. For a polar code of length N = 1024 and rate ¼, under DSCLF decoding with a list size L = 2 and a maximum number of trials $T_{\max}$ = 300, our proposed approach is shown to reduce the average execution time by 41.7% with four restart locations at the cost of approximately 1.5% in memory overhead.

## 1. Introduction

Polar codes [1] are a class of linear block codes that were shown to asymptotically achieve the channel capacity under low-complexity successive-cancellation (SC) decoding as the code length tends to infinity [1]. However, the SC decoding algorithm does not provide sufficient error-correction performance at short-to-moderate code lengths. The concatenation of a cyclic redundancy check (CRC) code to the polar code results in the CRC-aided (CA)–polar code scheme; this scheme was selected to protect the control channel of the enhanced mobile broadband (eMBB) service in 5G [2]. SC does not take advantage of the CRC code.

Successive-cancellation list (SCL) decoding [3] was proposed to improve the error-correction performance of SC decoding. SCL decoding retains *L* decoding paths, providing *L* different candidates of a codeword. To achieve this, the number of paths is doubled, and when it reaches 2L, the best *L* paths according to path metrics (PMs) are selected to continue the decoding. The true potential of the SCL decoding algorithm is highlighted with the CA–polar code scheme. The candidate with the smallest PM that verifies the CRC code is elected as the codeword estimate.

Another decoding algorithm for the CA–polar codes is the flip algorithm [4,5]. Unlike SCL decoding, successive-cancellation flip (SCF) and dynamic SCF (DSCF) sequentially attempt the decoding with a single SC instance, providing up to $T_{\max}$ candidates of a codeword. At each additional trial, one of the decision bits, based on a metric, is flipped during the course of SC decoding before normal decoding is resumed. DSCF better approximates the reliability of a decision, providing a more accurate list of bit-flipping candidates and improving the error-correction performance. Moreover, DSCF may also handle multiple bit flips per decoding trial. The latter requires a list of candidates that is dynamically updated along the decoding trials.

Successive-cancellation list flip (SCLF) decoding [6] combines the list and flip algorithms. Additional trials of SCLF correspond to the SCL algorithm having a reverse path selection on the path-flipping locations [7]. These locations are based on a flip metric computed during the initial SCL trial [8]. In [9], dynamic SCLF (DSCLF) decoding is proposed, which adapts the DSCF methodology to SCLF. That is, the metric that selects path-flipping locations is further improved, and the multi-path-flipping methodology of DSCF is adapted.

Since it combines list and flip algorithms, the algorithm is computationally complex and works according to the methodology outlined by [10,11,12,13,14,15], who investigated ways to reduce the complexity of the list–flip algorithm. In [10,11], a simplified metric and an adaptive list size *L* are proposed, allowing us to reduce the complexity of L≠2. A low-complexity flip metric allowing fast decoding of some special nodes is proposed in [12]. The fast implementation of the SCLF decoder is provided in [13], reducing up to 73.4% of the average decoding latency for N=512 and K=256 codes. In [14], the number of additional trials is restricted to one, but this specific case can be improved with a more accurate method for locating the first error position. In [15], the authors created a scheme with early termination, average execution time reduction, and enhanced performance by protecting the codeword with multiple CRC codes. However, the total number of CRC bits is high and specific patterns are required.

Previously, we proposed restart mechanisms for SCF-based decoders [16,17]. The simplified restart mechanism (SRM) conditionally restarts SC decoding from the second half of the codeword if that is where the current bit-flipping candidate is located. The generalized restart mechanism (GRM) restarts SC decoding from any location of the bit-flipping candidate, which is achieved by storing the decoded codeword in its memory at the end of the initial unsuccessful decoding trial. It was shown that GRM is applicable to various types of SCF-based decoders. This work represents the continuation of [16,17] but for the list–flip algorithm. The restart mechanism for the list–flip decoder shown in this paper reduces the complexity less in comparison to the GRM, and we will show that embedding the GRM is impracticable for this decoder. Nevertheless, the complexity reduction is better with respect to the SRM, and the error-correction performance is not reduced.

*Contributions*

This work proposes the limited-locations restart mechanism (LLRM) for SCLF-based decoders for polar codes with the central idea to partially skip decoding computations that are identical between the initial trial and any additional trial. For any additional trial, the decoding restart is performed from one of the restart locations that is the closest to the path-flipping location from its right-hand side (RHS) in a codeword. The restart locations are determined offline, i.e., by conducting simulations at the target frame-error rate (FER). An LLRM does not alter the error-correction performance of the original decoder. During a restart, the decoding tree is calculated from its root and partial-sum (PS) bits are recalculated. Relevant path information required to retrieve the decoding tree is stored to memory. When applying the LLRM with four restart locations to the DSCLF decoder that can flip up to three paths per decoding trial, the average flip time reduction is 51% for N=512 and K=128 while requiring approximately 1.7% of additional memory compared to the standard DSCLF decoder.

*Outline*

The remainder of this paper is organized as follows: Section 2 begins by introducing polar codes with SC decoding. Then, SCL, SCF, and SCLF decoders are described. Section 4 begins with a simulation-based statistical analysis that provides bit-flip location distributions. In Section 5, simulation results for SCLF and DSCLF decoders with LLRM, and previously proposed GRMs, for the standard scheme are provided. Comparisons are made in terms of error-correction performance, memory estimates, and average execution time.

## 2. Background

### 2.1. Construction of Polar Codes

An (N,K) polar code [1] is a linear block code of length $N=2^{n}$ and code dimension *K*, defining the code rate $R_{code}=K\;/\;N$. Polar codes encode an input vector $u=[u_{0},\ldots ,u_{N-1}]$ to the codeword $x=[x_{0},\ldots ,x_{N-1}]$ as $x=u{T_{2}}^{⊗n}$ where ${T_{2}}^{⊗n}$ denotes the Kronecker power of the binary kernel $T_{2}=\left[\begin{array}{cc} 1 & 0\\ 1 & 1 \end{array}\right]$. The matrix $T_{N}=T_{2}^{⊗n}$ induces channel polarization [1], i.e., each of the *N* bits u has its own bit channel defined by its own reliability. Classifying the *N* bit channels according to their reliabilities is not an easy task; several methods exist to achieve this in this paper, which can also be found in [18]. The polar code construction consists of splitting the *N* positions in u into two sets, A and $A^{c}$ (with |A|=K), being the information set and the frozen set. The information set corresponds to the indices of the *K* most reliable positions, while the remaining $\left(N-K\right)$ bits, called frozen bits, are set to predefined values that are known by the decoder, which are typically zeros. The vector u contains the message m of *K* bits in the positions stated in A, i.e., ${\hat{u}}_{A}=m$ and contains N−K frozen bits, i.e., ${\hat{u}}_{A^{c}}=0$. In this work, the binary phase-shift keying (BPSK) modulation over an additive white Gaussian noise (AWGN) channel is used, as well as the polar code construction of [18].

CA–polar codes include the concatenation of a CRC code during the polar encoding. The *r* CRC bits are generated on the basis of the message m and are placed in the set of information bits A, increasing the number of information bits to $K_{\mathrm{tot}}=K+r$. Next, information-bit indices of CA–polar codes are noted $a_{1}<\cdots <a_{K+r}$. The (N,K+r) notation is used throughout this work to indicate the code parameters of CA–polar codes.

### 2.2. SC Decoding

**Figure 1:**
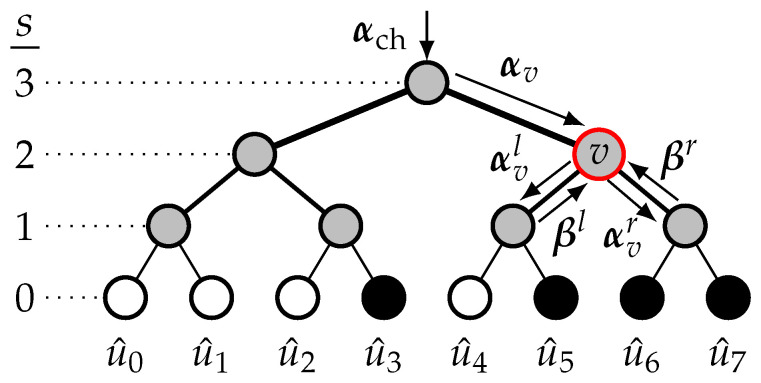
SC decoding tree of an $\left(8,4\right)$ polar code.

Polar codes have been proposed with the low-complexity SC algorithm in [1] to retrieve an estimate $\hat{u}$ of the input vector u. The description of SC decoding as a binary tree traversal was proposed in [19], where the tree is traversed depth-first, starting with the left branch. The decoding tree of an $\left(8,4\right)$ polar code is shown in Figure 1, where the stages are s∈{n,…,0} with the root node at s=n. The received vector of channel log-likelihood ratios (LLRs), denoted by $\alpha_{\mathrm{ch}}=\left[\alpha_{\mathrm{ch}}\left(0\right),\ldots ,\alpha_{\mathrm{ch}}\left(N-1\right)\right]$, is at the tree root. For any node, denoted by *v* and located at stage *s*, the input LLR vector is $\alpha_{v}=\left[\alpha_{v}\left(0\right),\ldots ,\alpha_{v}\left(2^{s}-1\right)\right]$ from the parent node, and the two input partial-sum (PS) vectors are $\beta^{l}=\left[\beta^{l}\left(0\right),\ldots ,\beta^{l}\left(2^{s-1}-1\right)\right]$ and $\beta^{r}=\left[\beta^{r}\left(0\right),\ldots ,\beta^{r}\left(2^{s-1}-1\right)\right]$, from its left and right child nodes, respectively. The left and right child nodes of the node *v* have the vectors $\alpha_{v}^{l}=\left[\alpha_{v}^{l}\left(0\right),\ldots ,\alpha_{v}^{l}\left(2^{s-1}-1\right)\right]$ and $\alpha_{v}^{r}=\left[\alpha_{v}^{r}\left(0\right),\ldots ,\alpha_{v}^{r}\left(2^{s-1}-1\right)\right]$, respectively, where each LLR element is calculated as follows:

(1)αvl(j)=fαv(j),αvj+2s−1,(2)αvr(j)=gαv(j),αvj+2s−1,βl(j),

where $j\in \{0,\;\ldots ,\;2^{s-1}-1\}$. Going left, the *f* function is calculated as [20]

(3) $$\begin{array}{c} f\left(\alpha_{1},\alpha_{2}\right)=\mathrm{sgn}\left(\alpha_{1}\right)\mathrm{sgn}\left(\alpha_{2}\right)\min\left(\left|\alpha_{1}\right|,\left|\alpha_{2}\right|\right). \end{array}$$

Going right, the *g* function is calculated as

(4) $$\begin{array}{c} g\left(\alpha_{1},\alpha_{2},\beta_{1}\right)=\left(1-2\beta_{1}\right)\alpha_{1}+\alpha_{2}. \end{array}$$

Bit estimates, $\hat{u}=\left[{\hat{u}}_{0},\ldots ,{\hat{u}}_{N-1}\right]$, are obtained either by taking a hard decision on the decision LLRs, $\alpha_{\mathrm{dec}}=\left[\alpha_{\mathrm{dec}}\left(0\right),\ldots ,\alpha_{\mathrm{dec}}\left(N-1\right)\right]$, that reach the leaf nodes or using the frozen bit values. Namely, we have

(5) $${\hat{u}}_{i}=\left\{\begin{array}{cc} \mathrm{HD}\left(\alpha_{\mathrm{dec}}\left(i\right)\right),\;\; & \mathrm{if}\;i\in A\\ 0, & \mathrm{if}\;i\in A^{c}. \end{array}\right.$$

where HD(·) represents the hard decision function. In Figure 1, the nodes represented in black and white correspond to information and frozen bits. The partial-sum vector, denoted by β, is calculated from the bit estimates and is propagated up from children to parent nodes. At any node *v*, each bit of β is calculated as follows:

(6) $$\beta_{v}\left(j\right)=\left\{\begin{array}{c} \beta^{l}\left(j\right)⊕\beta^{r}\left(j\right),\;\;\;\mathrm{if}\;\;j<2^{s-1},\\ \beta^{r}\left(j\right),\;\;\;\;\;\;\;\;\;\mathrm{otherwise}, \end{array}\right.$$

where $j\in \{0,\ldots ,2^{s}-1\}$ and ⊕ is a bitwise XOR.

### 2.3. SCL Decoding

SCL decoding was proposed in [3] and uses *L* parallel instances of the SC decoder, where *L* is a power of 2. During the decoding, the *L* best decoding paths are kept through the computation of PMs. At the end of the decoding, a set of *L* bit-estimate candidates, denoted as ${\hat{U}}_{N-1}=\left\{\hat{u}\left(1\right),\ldots ,\hat{u}\left(L\right)\right\}$, is provided. Next, the SCL decoding is described in more detail.

First, for all paths, the bit-estimate vectors are initialized to 0, i.e., $\forall l\in \left[1,L\right],\hat{u}\left(l\right)=0$, with *l* denoting the path index. Moreover, all PMs are also initialized to 0, i.e., ∀l∈[1,L],PM(l)=0. The decoding starts with a single path, and intermediate LLRs are computed according to the SC schedule and the update rules (1) and (2). When the decoder reaches the first information bit $a_{1}$, the single path is duplicated, i.e., all intermediate partial sums and all intermediate LLRs including the decision LLR $\alpha_{\mathrm{dec}}\left(a_{1}\right)$ are duplicated to another path structure. Contrary to SC decoding where the bit-estimate ${\hat{u}}_{a_{1}}$ is taken according to the hard decision $\mathrm{HD}\left(\alpha_{\mathrm{dec}}\left(a_{1}\right)\right)$ (5), the path duplication in SCL decoding allows us to consider both options {0,1} regardless of the sign of the LLR $\alpha_{\mathrm{dec}}\left(a_{1}\right)$. However, the PMs are updated based on the chosen bit decision and the sign of the decision LLR $\alpha_{\mathrm{dec}}$ on their path. Namely, the PM is penalized if the bit decision does not follow the sign of the decision LLR, i.e., for the *l*-th path, the path metric PM(l) is updated as [21]

(7) $$\begin{array}{c} \mathrm{PM}\left(l\right)=\left\{\begin{array}{cc} \mathrm{PM}\left(l\right)+\left|\alpha_{\mathrm{dec}}\left(l,a\right)\right|, & \mathrm{if}\;\;\mathrm{HD}\left(\alpha_{\mathrm{dec}}\left(l,a\right)\right)\neq {\hat{u}}_{a}\left(l\right)\\ \mathrm{PM}\left(l\right),\; & \mathrm{otherwise}, \end{array}\right. \end{array}$$

where $\alpha_{\mathrm{dec}}\left(l,a\right)$ is the decision LLR for the *l*-th path at index a∈A, while ${\hat{u}}_{a}\left(l\right)$ is the bit decision.

For the first information indices $A_{\mathrm{dup}}≜\left\{a_{1},\ldots ,a_{log_{2}\left(L\right)}\right\}$, the duplication of the paths does not result in more than *L* paths; hence, no sorting and selection of the best *L* paths are required. However, for all information bits $a\in A_{\mathrm{sort}}≜\left\{a_{log_{2}\left(L\right)+1},\ldots ,a_{K+r}\right\}$, 2L paths are obtained after duplication. Hence, a selection of the best *L* paths is required and is performed through the sorting operation of the 2L PMs. The *L* paths having the smallest PM remain.

After duplication, the set of 2L PMs is noted ${\mathrm{PM}}_{{\mathrm{dup}}_{a}}=\left\{\mathrm{PM}\left(1\right),\ldots ,\mathrm{PM}\left(2L\right)\right\}$ with $a\in A_{\mathrm{sort}}$, $\forall \;\mathrm{PM}\left(l\right)\in {\mathrm{PM}}_{{\mathrm{dup}}_{a}}$, and PM(l) is updated as (7). For $\forall \;a\in A_{\mathrm{sort}}$, the set ${\hat{U}}_{{\mathrm{dup}}_{a}}=\left\{{\hat{u}}_{0}^{a}\left(1\right),\ldots ,{\hat{u}}_{0}^{a}\left(2L\right)\right\}$ gathers the 2L partial candidates after duplication at index *a*. After the sorting of ${\mathrm{PM}}_{{\mathrm{dup}}_{a}}$, the following notations are used: ${\mathrm{PM}}_{{\mathrm{sort}}_{a}}=\left\{{\mathrm{PM}}_{a},{\mathrm{PM}}_{{\mathrm{worst}}_{a}}\right\}$ and ${\hat{U}}_{{\mathrm{sort}}_{a}}=\left\{{\hat{U}}_{a},{\hat{U}}_{{\mathrm{worst}}_{a}}\right\}$. Each subset contains the information of *L* paths. The SCL decoding continues with the best *L* paths composed of the partial bit estimates ${\hat{U}}_{a}=\left\{{\hat{u}}_{0}^{a}\left(1\right),\ldots ,{\hat{u}}_{0}^{a}\left(L\right)\right\}$ and the corresponding *L* PMs ${\mathrm{PM}}_{a}=\left\{\mathrm{PM}\left(1\right),\ldots ,\mathrm{PM}\left(L\right)\right\}$. For $i\in A^{c}$, the *L* paths takes the frozen decision (5) and the update of the *L* PMs is performed according to (7).

SCL decoding shows its true potential for CA–polar codes. At the end of the decoding, a CRC check is performed on the *L* candidates stored in ${\hat{U}}_{N-1}$. The final decoded codeword is the candidate that satisfies the CRC while minimizing the path metric. If none of the bit estimates satisfy the CRC, a decoding failure is declared.

### 2.4. SCF Decoding

The SCF algorithm was proposed in [4] for CA–polar codes. This algorithm reuses a single SC instance to perform the decoding. If the CRC check fails at the end of the initial SC decoding trial, up to $T_{\max}$ additional trials are performed, each of them involving a bit flip in the bit-estimate $\hat{u}$. The decoding stops when one additional trial returns a candidate $\hat{u}$ checking the CRC code or after $T_{\max}$ additional trials resulting in a CRC check failure. The DSCF decoding was proposed to enhance the performance of SCF [5]; DSCF is defined by the decoding order ω, stating the maximum number of bit flips that can be performed in the additional trial. If ω=1, the list of bit-flip candidates is known at the end of the initial trial; if ω={2,3}, the list of bit-flip candidates is adjusted in all additional trials, i.e., the list is dynamic. A greater decoding order ω results in greater error-correction gain but also requires more $T_{\max}$ trials as well as more memory because of the storage of the flip metrics and of the bit-flip locations. For the *t*-th additional trial, $1\leq t\leq T_{\max}$, the set $\varepsilon_{t}$ stores the bit-flip locations with $|\varepsilon_{t}|\leq \omega$. The list of all bit-flipping candidates is denoted as $B_{\mathrm{flip}}=\left\{\varepsilon_{1},\ldots ,\varepsilon_{t},\ldots \varepsilon_{T_{\max}}\right\}$.

For ω≥2, the latency of DSCF is its main drawback due to the sequential trials and the greater $T_{\max}$ to approach the full potential of the decoding algorithm. Options exist to improve the decoding latency of DSCF. The baseline algorithm can be the fast-SSC algorithm [22], having special nodes in order to not traverse the full decoding tree. However, for ω≥2, the special nodes need to be adapted and reduced in size, leading to a more complex implementation [23] with respect to ω=1. Another option is the restart mechanism [16,17].

*An optimal restart mechanism for flip decoders: the generalized restart mechanism (GRM):*

**Figure 2:**
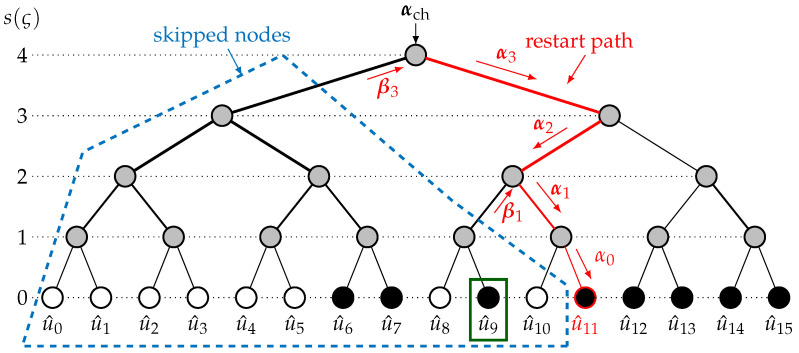
Additional trial for flip decoders embedding the GRM; the first bit flip is $\min(\varepsilon_{t})=9$ and the restart location is $\psi_{t}=11$.

Authors in [17] proposed the GRM to restart the decoding elsewhere other than the first leaf for the additional trials. At additional trial $1\leq t\leq T_{\max}$, the restart location $\psi_{t}$ depends on the information set A and the first bit-flip location $\min(\varepsilon_{t})$. This mechanism requires only the storage of $\hat{u}$, leading to a small memory overhead that allows the retrieval of the intermediate partial sum $\beta_{\mathrm{int}}$. A module to perform the correct *f* and *g* sequence is required to retrieve the intermediate LLRs $\alpha_{\mathrm{int}}$. This sequence is retrieved with the binary representation of $\psi_{t}$ [17]. Figure 2 depicts the additional trial for a flip decoder embedding the GRM. The first bit-flip location is $\min(\varepsilon_{t})=9$, leading to the restart location $\psi_{t}=11$. The skipped nodes are shown in blue, while the restart path is depicted in red. The GRM can be embedded into any baseline algorithm, i.e., standard SC [1] or the fast-SSC [22] decoding algorithm. The average reduction brought by the GRM depends on the code rate and the baseline algorithm. For N=1024 and code rate $R_{code}=$ ⅛, the reduction is up to 56.9% with SC as the baseline algorithm and 20.9% with fast-SSC as the baseline algorithm.

### 2.5. SCLF Decoding

SCLF decoding is a combination of list decoding [3] and flip decoding [4,5]. It was first proposed in [6], but the baseline algorithm described in [8] is used in this paper. The least reliable bit positions, called *path-flipping locations*, are identified thanks to the flip metrics computed during the initial SCL trial and are stored in $B_{\mathrm{flip}}$. For $a\in A_{\mathrm{sort}}$, the flip metric ${\mathrm{FM}}_{a}$ in [8] is computed using the 2L PMs after the sorting operation ${\mathrm{PM}}_{{\mathrm{sort}}_{a}}$ as

(8) $${\mathrm{FM}}_{a}=\ln\left(\frac{\sum_{l=0}^{L-1}\exp\left(-\mathrm{PM}\left(l\right)\right)}{{\left(\sum_{ł=0}^{L-1}\exp\left(-\mathrm{PM}\left(l+L\right)\right)\right)}^{p}}\right),$$

where ln(·) indicates the natural logarithm. The constant value p≈1.0 is found via simulations. A simplified metric of (8) has been proposed in [12] and is computed as follows:

(9) $${\mathrm{FM}}_{a}=-\mathrm{PM}\left(1\right)+p\cdot \mathrm{PM}\left(L+1\right),$$

where PM(1) represents the best metric while PM(L+1) is the best metric among the paths that will be discarded at index $a\in A_{\mathrm{sort}}$. The use of (9), which has also been applied to partitioned SCLF (PSCLF) decoding [15], leads to a negligible error-correction performance degradation. Thus, the metric (9) is used in this work for SCLF decoding. During the initial trial, $|A_{\mathrm{sort}}|$ flip metrics were computed. The metrics are sorted when computed while keeping track of the corresponding index $a\in A_{\mathrm{sort}}$. The $T_{\max}$ smallest flip metrics are stored in the set $M_{\mathrm{flip}}$. The $T_{\max}$ corresponding indices are stored in $B_{\mathrm{flip}}=\left\{\varepsilon_{1},\ldots ,\varepsilon_{T_{\max}}\right\}$ where $\varepsilon_{t}$ corresponds to the path-flipping location in the *t*-th additional trial of SCLF.

In SCLF, $|\varepsilon_{t}|=1$, i.e., the path-flipping only occurs once per additional trial. During the additional trial *t*, the standard SCL trial is performed until the path-flipping location $\varepsilon_{t}$ is reached. At this position, the *L* worst paths are selected instead of the *L* best ones, i.e., *path flipping* is performed [7]. Following the path flipping, the standard SCL decoding is resumed for the remaining part of the codeword.

### 2.6. Dynamic SCLF Decoding

In [9], the DSCF decoding strategy is adapted to SCLF decoding, and DSCLF-ω decoding is proposed where several path-flipping locations potentially occur in an additional trial. The metric computations of the DSCLF and DSCF decoders are very similar. $B_{\mathrm{flip}}$ is dynamically updated for each unsuccessful additional trial and is then composed of a set of path-flipping candidates. For the *t*-th additional trial, the path-flipping candidate $\varepsilon_{t}$ then becomes a set with $|\varepsilon_{t}|\leq \omega$. During the *t*-th additional trial, if $|\varepsilon_{t}|<\omega$, for all information indices $a>\max(\varepsilon_{t})$, a flip metric is computed for the extended set $\varepsilon_{t}\cup a$ and the metric is calculated as follows [9]:

(10) $${\mathrm{FM}}_{\varepsilon_{t}\cup a}=\sum_{\epsilon \in \varepsilon_{t}\cup a}{\mathrm{FM}}_{\epsilon }+\sum_{\begin{array}{c} j\leq a\\ j\in A \end{array}}J\left({\mathrm{FM}}_{j}\right),$$

where ${\mathrm{FM}}_{j}$ corresponds to the simplified metric (9) and $J\left(x\right)$ is calculated as follows [5,9]:

(11) $$J\left(x\right)=\frac{1}{z}\ln\left(1+\exp\left(-z\cdot x\right)\right),$$

where *z* is a constant value, at 0.0<z≤1.0, and is found via simulations. In [10], the piece-wise linear approximation function of (10) is proposed. In [5], a step-approximation function of (10) is derived for the DSCF decoder, and it is calculated as follows:

(12) $$J_{\mathrm{step}}\left(x\right)=\left\{\begin{array}{c} 1.5\;,\;\mathrm{if}\;\;0\leq x\leq 5.0,\\ 0\;,\;\;\;\;\mathrm{otherwise}. \end{array}\right.$$

For DSCLF-ω, ω≥2, during an additional trial, several flip metrics (10) are potentially computed; as soon as one flip metric is computed, sorting is performed to update $B_{\mathrm{flip}}$ and $M_{\mathrm{flip}}$ of size $T_{\max}$. If the metric ${\mathrm{FM}}_{\varepsilon_{t}\cup a}$ exceeds the largest metric in $M_{\mathrm{flip}}$, no sorting is required, and the set $\varepsilon_{t}\cup a$ is discarded. If not, the set $\varepsilon_{t}\cup a$ is inserted to $B_{\mathrm{flip}}$ and ${\mathrm{FM}}_{\varepsilon_{t}\cup a}$ is inserted in $M_{\mathrm{flip}}$ while maintaining the ascending order.

To the best of our knowledge, the use of (9) in the dynamic metric of DSCLF (10) using the approximation (12) has never been investigated in previous works. This combination makes a low complexity flip metric for the DSCLF decoding algorithm. However, the main focus of this paper is on a restart mechanism to reduce the average execution time; hence, no more comments on this reduced complexity flip metric will be made later on.

## 3. Restart Mechanism for the List-Flip Decoder

In [17], the GRM showed great potential in reducing the complexity and the average execution time of flip-based decoders, i.e., SCF and DSCF-ω, regardless of the baseline algorithm (with or without fast decoding of special nodes). The GRM only requires the storage of the candidate $\hat{u}$ of the initial SC trial to enable the restart of an additional trial in any location a∈A. Next, the feasibility of embedding the GRM to the SCLF algorithm is discussed based on the memory overhead required. Then, a restart mechanism tailored for the list–flip algorithm is proposed and described.

### 3.1. Memory Requirements of List-Flip Algorithm

First, the memory requirements of SCLF are studied. The SCL decoder uses a structure of *N* bits to store A. The SCLF (DSCLF-ω) decoder corresponds to an SCL decoder, used up to $T_{\max}$ times, an additional module to compute the flip metric (9) and (10), and a module to construct $B_{\mathrm{flip}}$. The SCL decoder is considered to be parallel and is viewed as *L* SC instances in terms of memory. The memory requirements, expressed in bits, for the LLRs and the partial sums of an SC instance is described in [17] and is

(13) $$\begin{array}{c} \Lambda_{\mathrm{SC}}=\underset{\alpha_{\mathrm{ch}}}{\underset{︸}{Q_{\mathrm{ch}}\cdot N}}+\underset{\alpha_{\mathrm{int}}}{\underset{︸}{Q_{\mathrm{int}}\cdot \left(N-1\right)}}+\underset{\hat{u}+\beta_{\mathrm{int}}}{\underset{︸}{2N-1}}, \end{array}$$

where $Q_{\mathrm{ch}}$ and $Q_{\mathrm{int}}$ represent the quantization in bits for the channel LLRs $\alpha_{\mathrm{ch}}$ and the intermediate LLRs $\alpha_{\mathrm{int}}$. *N* bits are used to store the current candidate $\hat{u}$ and N−1 bits are needed for the intermediate partial sums $\beta_{\mathrm{int}}$. The SCL algorithm requires the storage of the structure ${\mathrm{PM}}_{\mathrm{dup}}$, i.e.,

(14) $$\begin{array}{c} \Lambda_{\mathrm{SCL}}=L\cdot \Lambda_{\mathrm{SC}}+\underset{A}{\underset{︸}{N}}+\underset{{\mathrm{PM}}_{\mathrm{dup}}}{\underset{︸}{2L\cdot Q_{\mathrm{PM}}}}, \end{array}$$

where $Q_{\mathrm{PM}}$ is the quantization in bits used for the PMs.

Regarding the flip algorithm, DSCLF-ω requires the storage of $B_{\mathrm{flip}}$ and $M_{\mathrm{flip}}$ leading to

(15) $$\begin{array}{c} \Lambda_{\mathrm{flip}}=\underset{M_{\mathrm{flip}}}{\underset{︸}{T_{\max}\cdot Q_{\mathrm{flip}}}}+\underset{B_{\mathrm{flip}}}{\underset{︸}{\omega \cdot T_{\max}}}, \end{array}$$

where $Q_{\mathrm{flip}}$ is the quantization in bits used for the flip metric in (9) and (10). In bits, the memory of the list–flip decoder is

(16) $$\begin{array}{c} \Lambda_{\mathrm{list}\text{-}\mathrm{flip}}=\Lambda_{\mathrm{SCL}}+\Lambda_{\mathrm{flip}}. \end{array}$$

### 3.2. Generalized Restart Mechanism

The GRM is a restart mechanism that reduces the complexity of flip-based decoders while not affecting the error-correction performance [17]. The small memory overhead used to embed the GRM is one of the main advantages of this mechanism, allowing a reduction of 56.9% for the average execution time at the cost of 3.4% memory overhead for DSCF-3 decoding of a (1024,128+11) polar code. Another key advantage is that the restart location for an additional trial is always optimal, as the set of restart locations in the GRM is defined as $R_{\mathrm{GRM}}=A_{\mathrm{sort}}$. Next, the estimation of the memory overhead is performed to embed the GRM to SCLF.

At the additional trial $1\leq t\leq T_{\max}$, the GRM embedded into the flip decoder allows us to restart the decoding at the restart location $\psi_{t}\in A_{\mathrm{sort}}$, which is the next information-bit location after $\min\left(\varepsilon_{t}\right)\in A_{\mathrm{sort}}$. The restart path retrieves the intermediate partial sum $\beta_{\mathrm{int}}$ based on the $\hat{u}$ of the initial SC trial. The intermediate LLRs, $\alpha_{\mathrm{int}}$, are retrieved with the channel LLRs, $\alpha_{\mathrm{ch}}$, and the intermediate partial sum $\beta_{\mathrm{int}}$. The sequence of *f* and *g* functions to perform during the restart path depends on the binary representation of $\psi_{t}$ as described in [17]. Hence, the restart path allows us to restore the status of the tree at position $\psi_{t}$ without performing the usual SC schedule. Next, we describe why the SCL cannot be restarted with only the storage of the final candidate ${\hat{U}}_{N-1}$.

At a path-flipping location, the *L* worst paths instead of the *L* best continue the decoding. The *L* worst path information, i.e., the sets ${\mathrm{PM}}_{{\mathrm{worst}}_{a}}$ and ${\hat{U}}_{{\mathrm{worst}}_{a}}$, changes at each information bit $a\in A_{\mathrm{sort}}$ and becomes overwritten during the initial SCL trial. At a certain position, if the best *L* paths out of the 2L are generated through less than *L* parents, the information carried of some discarded paths is lost and cannot be retrieved with ${\hat{U}}_{N-1}$.

As an example, SCLF with L=2 decoding an (8,3+1) code with A={3,5,6,7} ($A_{\mathrm{sort}}=\left\{5,6,7\right\}$) is shown. In this example, only the bit values at position A are shown. At position 3, SCL considers L=2 paths, i.e., ${\hat{U}}_{3}=\left\{\left[0\right],\left[1\right]\right\}$. After duplication at position 5, we have ${\hat{U}}_{{\mathrm{dup}}_{5}}=\left\{\left[0,0\right],\left[0,1\right],\left[1,0\right],\left[1,1\right]\right\}$, which, after sorting, will be divided as ${\hat{U}}_{5}=\left\{\left[0,0\right],\left[0,1\right]\right\}$ and

(17) $$\begin{array}{c} {\hat{U}}_{{\mathrm{worst}}_{5}}=\left\{\left[1,0\right],\left[1,1\right]\right\}. \end{array}$$

If we forward to the end with ${\hat{U}}_{7}=\left\{\left[0,0,1,0\right],\left[0,0,0,1\right]\right\}$, this is not able to pass the CRC. Only storing ${\hat{U}}_{7}$ will not be enough to retrieve the worst paths at position 5 for example. Indeed, from ${\hat{U}}_{7}$, we can state that $\left\{\left[0,0\right]\right\}\subset {\hat{U}}_{5}$, meaning that ${\hat{U}}_{{\mathrm{worst}}_{5}}\subset \left\{\left[0,1\right],\left[1,0\right],\left[1,1\right]\right\}$, without knowing ${\hat{U}}_{{\mathrm{worst}}_{5}}$ (17), which is not enough information to perform the path flipping through the GRM.

Hence, in order to resume the decoding in any path-flipping location $\min(\varepsilon_{t})$, the *L* worst path information, i.e., ${\hat{U}}_{{\mathrm{worst}}_{a}}$ and ${\mathrm{PM}}_{{\mathrm{worst}}_{a}}$, need to be stored for all $a\in A_{\mathrm{sort}}$. In order to store the path metric information, $L\times Q_{\mathrm{PM}}\times \left|A_{\mathrm{sort}}\right|$ additional bits are required. In order to save memory for the storage of the *L* worst partial candidate ${\hat{U}}_{{\mathrm{worst}}_{a}}$, only the message bits can be stored, i.e., for $a_{i}\in A_{\mathrm{sort}}$, L·i bits are stored instead of $L\cdot a_{i}$. Hence, the total memory requirement $\Lambda_{\mathrm{GRM}}$ for embedding the GRM to the list–flip algorithms is

(18)ΛGRM=∑i=log2(L)+1K+rPMworst+L·i,(19)ΛGRM=L×QPM·|Asort|+∑i=log2(L)+1K+ri.

**Figure 3:**
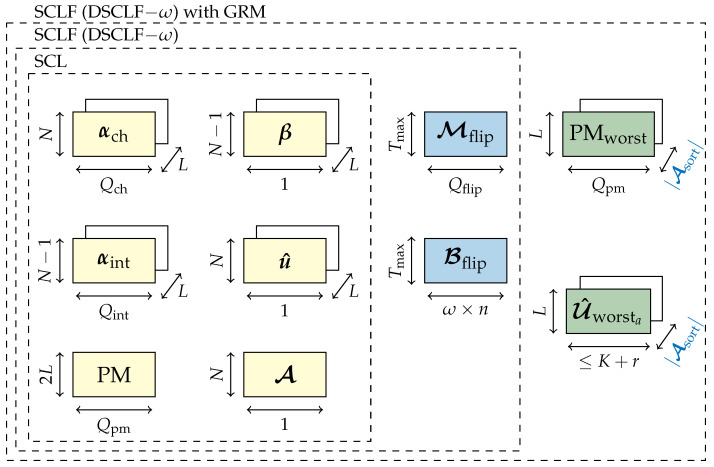
Memory sketch of SCLF with GRM.

The memory requirement (19) grows with the code rate since $|A_{\mathrm{sort}}|=K+r-\log2\left(L\right)$ will grow as well. Moreover, the memory will grow if the list size grows (19). Figure 3 depicts the memory sketch of the DSCLF-ω algorithm embedding the GRM.

The storage of the *L* worst partial candidates leads to impractically large memory requirements. As an example, for the list–flip decoder of the (1024,512+16) polar code with L=2,ω=2, and $T_{\max}=50$ and the quantization scheme $Q_{\mathrm{ch}}=6$, $Q_{\mathrm{int}}=7$, $Q_{\mathrm{PM}}=8$, and $Q_{\mathrm{flip}}=9$, derived from [16,23], the memory requirement of the decoder $\Lambda_{\mathrm{list}\text{-}\mathrm{flip}}$ and embedding the GRM $\Lambda_{\mathrm{GRM}}$ are estimated to be

(20)Λlist-flip=31760bits,(21)ΛGRM=287758bits.

Hence, the GRM induces a memory overhead of $\Delta_{\mathrm{mem}}^{\mathrm{GRM}}=906\%$, while it was 6.1% for DSCF-2 with the same $T_{\max}$ [17].

The memory requirements to embed the GRM to list–flip algorithms, i.e., having $R_{\mathrm{GRM}}=A_{\mathrm{sort}}$, has been estimated with the conclusion that the GRM is unfeasible for the list–flip decoder. Next, the LLRM is proposed to tackle this issue.

### 3.3. Limited Location Restart Mechanism

The LLRM is derived from the GRM and proposed as a way to skip part of the tree traversal of the SCL decoding. However, the set of restart locations R is predefined and its size |R| is very limited compared to $|R_{\mathrm{GRM}}|$. The effectiveness of the proposed approach is discussed in Section 5. First, the proposed LLRM is defined.

For an additional trial *t*, the list–flip decoder embedding the proposed LLRM skips non-negligible parts of the decoding tree by recovering the path information at the location $\psi_{t}\in R$, where $R=\left\{R\left(1\right),\ldots ,R\left(R\right)\right\}\subset A_{\mathrm{sort}}$ represents the set of restart locations and R=|R| is the number of restart locations. It should be noted that $a_{\log_{2}\left(L\right)+1}\in A_{\mathrm{sort}}$ will always be included in R. A notation variant of R is the subset $R_{A}=\left\{R_{A}\left(1\right),\ldots ,R_{A}\left(R\right)\right\}\subset \left[\log_{2}\left(L\right)+1,K+r\right]$, storing the index of each restart location in the information set A, i.e., $R=\left\{a_{R_{A}\left(1\right)},\ldots ,a_{R_{A}\left(R\right)}\right\}$.

By embedding the proposed LLRM, the SCL additional trial is modified by not restarting at position 0. The corresponding modified SCL trial is denoted by $\mathrm{SCL}\left(\psi_{t},\varepsilon_{t}\right)$, indicating the restart location $\psi_{t}\in R$ and the bit-flipping set $\varepsilon_{t}$. The savings in terms of computations and decoding time come at the cost of storing all path information in the positions stated in R during the initial SCL trial. As described next, restart mechanisms exist [16,24] for SCF (DSCF-ω) but on positions permitting an easy restart procedure.

In [16], the simplified restart mechanism (SRM) was proposed and can be seen as an instance of LLRM with one of the most simple sets of restart locations R. In the SRM, R=2 restart locations are possible and include $R_{\mathrm{SRM}}=\left\{0,\frac{N}{2}\right\}$ which allows a very simple restart path, where the restart always begins from the upper stage involving the channel LLRs $\alpha_{\mathrm{ch}}$. Only $\frac{N}{2}$ bits are required to apply this mechanism. However, the restart location $R_{\mathrm{SRM}}\left(1\right)=0$ does not allow a reduction in the complexity. Hence, only the restart location $R_{\mathrm{SRM}}\left(2\right)=\frac{N}{2}$ reduces the decoding complexity. The authors in [24] derive the SRM to propose the set of restart locations $R_{\mathrm{FRM}}=\left\{0,\frac{N}{2},\frac{3N}{4},\ldots ,\frac{(N-1)N}{N}=N-1\right\}$. Despite being a more advanced set of restart locations, it comes at the cost of storing LLRs. As for the SRM, the restart locations are only on the right-hand side of the tree, limiting the application of the mechanism as discussed in [16].

The optimal restart mechanism is the GRM [17] as it combines the ability of restarting at any location in A, i.e., $R_{\mathrm{GRM}}=A$, while not having to store the intermediate LLRs. This is possible thanks to the *generalized restart path*, which describes the algorithm that restores all intermediate LLRs and all partial sums on the basis of the binary representation of the restart location $\psi_{t}\in A$ and on the SC candidate $\hat{u}$ of the initial trial.

By reusing the generalized restart path, the restart locations in the proposed LLRM will not be selected according to the ease of performing the restart path as in [16,24] but on picking the restart locations to improve, as much as possible, the average execution time reduction. The first consequence is that the first restart location for the proposed LLRM is not 0 but will be $R\left(1\right)=a_{\log_{2}\left(L\right)+1}\in A_{\mathrm{sort}}$ ($R_{A}\left(1\right)=\log_{2}\left(L\right)+1$), representing the first information-bit location involving duplication in SCL. This will allow the avoidance of computations on the left-hand side of the decoding tree mostly composed of frozen bits. However, before finding the other restart locations, the proposed LLRM is described in more detail.

The memory overhead of the LLRM is first discussed. It is decomposed into two sets: the set P={P(1),…,P(R)} that will store the relevant PMs and the set $\hat{M}=\left\{\hat{M}\left(1\right),\ldots ,\hat{M}\left(R\right)\right\}$ that will store the relevant messages. During the initial SCL trial, when the decoding reaches the first restart location R(1)∈R, the 2L-sorted PMs are stored in P(1), while the partial message candidates are stored in $\hat{M}\left(1\right)$. For the other restart locations, path information is also stored in the corresponding index of P and $\hat{M}$. At the end of the initial SCL trial, the *R* elements of P and $\hat{M}$ gather the full path information at the restart locations stored in the R of the *L* best and *L* worst paths.

The LLRM is activated whenever an additional trial is performed, i.e., if the CRC check fails for all *L* candidates in ${\hat{U}}_{N-1}$. For $1\leq t\leq T_{\max}$, the *t*-th additional trial is defined by the path-flipping locations stored in $B_{\mathrm{flip}}\left(t\right)=\varepsilon_{t}$. The first path-flipping location is defined by $\min(\varepsilon_{t})$. The restart location $\psi_{t}\in R$ for the *t*-th additional trial corresponds to the closest element in R, verifying that

(22) $$\begin{array}{c} \psi_{t}\leq \min\left(\varepsilon_{t}\right). \end{array}$$

The additional SCL trial restarting at $\psi_{t}$ and flipping at locations stored in $\varepsilon_{t}$ is noted for $\mathrm{SCL}\left(\psi_{t},\varepsilon_{t}\right)$. Next, ρ denotes the index in R, defining $\psi_{t}$, i.e., $\psi_{t}=R\left(\rho \right)$.

If $\psi_{t}=\min\left(\varepsilon_{t}\right)$, the restart location is also a path-flipping location; hence, the worst *L* paths are chosen to continue the decoding. Thus, the path metric structure ${\mathrm{PM}}_{\psi_{t}}$ of $\mathrm{SCL}\left(\psi_{t},\varepsilon_{t}\right)$ is restored with the *L* worst PMs stored in P(ρ). Similarly, the partial candidate ${\hat{U}}_{\psi_{t}}$ is restored on the basis of the *L* worst partial candidates stored in P(ρ). If $\min\left(\varepsilon_{t}\right)>\psi_{t}$, the restart location precedes the path-flipping location; hence, the best *L* paths are chosen to continue the decoding. Thus, the path metric structure ${\mathrm{PM}}_{\psi_{t}}$ of $\mathrm{SCL}\left(\psi_{t},\varepsilon_{t}\right)$ is restored with the best path metric stored in P(ρ). Similarly, the partial candidate ${\hat{U}}_{\psi_{t}}$ is restored on the basis of the *L* best partial candidates stored in P(ρ).

Once the structures ${\mathrm{PM}}_{\psi_{t}}$ and ${\hat{U}}_{\psi_{t}}$ are correctly restored, for all *L* paths, the intermediate values in the decoding tree, i.e., the restart path, are used to attain the next leaf in the decoding trees of the *L* paths, i.e., $\psi_{t}+1$, as shown in Figure 2. The binary representation of $\psi_{t}+1$ describes the series of functions to be performed in the restart path. If *g*-functions are required, the partial sum $\beta_{\mathrm{int}}$ is restored through the *L*-independent restart paths. These functions allow us to accurately retrieve the intermediate LLRs $\alpha_{\mathrm{int}}$ for all *L* paths. When the *L* restart paths attain the leaf, if $\psi_{t}+1\in A^{c}$, for all *L* paths, ${\hat{u}}_{\psi_{t}+1}=0$ and the *L* path metrics are updated using (7). If $\psi_{t}+1\in A$, 2L paths are generated, as well as 2L path metrics, and the decision on the 2L paths are taken based on the path metrics and the nature of position $\psi_{t}+1$.

### 3.4. Example of the LLRM

**Figure 4:**
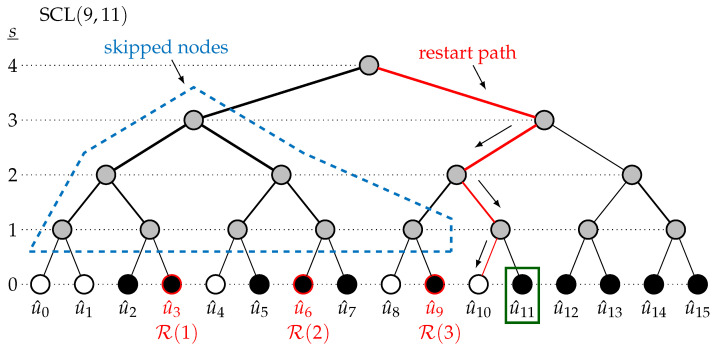
The modified trial SCL (9,11) with R={3,6,9}.

The modified SCL trial in SCLF embedding the LLRM, $\mathrm{SCL}\left(\psi_{t},\varepsilon_{t}\right)$, is explained with an example for the $\left(16,10+1\right)$ code defined by A={2,3,5,6,7,9,11,12,13,14,15} with $T_{\max}=1$ and L=2. The set of restart locations is set prior to decoding to R={3,6,9}. This example is shown in Figure 4. The initial trial starts with P={∅,∅,∅} and $\hat{M}=\left\{\emptyset ,\emptyset ,\emptyset \right\}$. When reaching the first information bit $a_{1}=2$, the decoding considers both ${\hat{u}}_{a_{1}}=\left\{0,1\right\}$, leading to the structure ${\hat{U}}_{a_{1}}=\left\{\left[0,0,0\right],\left[0,0,1\right]\right\}$. The SCL does not need sorting since L=2. The path metric structure is also updated as ${\mathrm{PM}}_{a_{1}}=\left\{\mathrm{PM}\left(1\right),\mathrm{PM}\left(2\right)\right\}$. The next bit $a_{2}=3\in A$ is also R(1). As explained in Section 3.3, $\hat{M}\left(1\right)$ will store the 2L partial candidates, i.e., $\hat{M}\left(1\right)=\left\{\left[0,0\right],\left[0,1\right],\left[1,0\right],\left[1,1\right]\right\}$, before the selection. Similarly, the 2L PMs in ${\mathrm{PM}}_{{\mathrm{sort}}_{a1}}$ are stored in P(1). The same storage process happens when the decoding reaches R(2)=6 and R(3)=9.

After the failure of the initial trial, the path-flipping location is considered to be $\varepsilon_{1}=\left\{11\right\}$ in this example. The restart location $\psi_{1}$ is selected from R as being the closest to $\varepsilon_{1}$, verifying $\psi_{1}\leq \varepsilon_{1}$. Hence, the restart location is $\psi_{1}=R\left(3\right)=9$. The $\mathrm{SCL}\left(\psi_{1},\varepsilon_{1}\right)$ begins by restoring ${\hat{U}}_{9}$ and ${\mathrm{PM}}_{9}$ with the *L* best partial candidates in $\hat{M}\left(3\right)$ and the *L* best PMs in P(3) since $\psi_{1}\neq \varepsilon_{1}$. For all *L* paths, the restart path connecting the root of the tree at stage s=n=4 with the leaf ${\hat{u}}_{10}$ at stage s=0 is traversed, which allows us to retrieve the intermediate partial sum $\beta_{\mathrm{int}}$ and the intermediate LLR $\alpha_{\mathrm{int}}$. After traversing the restart path, all intermediate information is retrieved, allowing us to resume the standard course of SCL; since $10\in A^{c}$, only the *L* path metrics are updated based on the decision LLR computed at the end of the restart path for the *L* paths. When $\varepsilon_{1}=11$ is reached, path flipping is performed, i.e., the *L* worst paths are picked. When the last index is reached, the CRC is verified on all decoding paths. It either returns a decoding candidate verifying the CRC or returns a decoding failure since the decoding has reached its maximum number of additional trials $T_{\max}=1$.

### 3.5. Memory Model

In order to resume the decoding in one of the *R* restart locations, the LLRM requires the storage of

1. the set of restart locations R;
2. the 2L path metric information P on each restart location R(ρ)∈R;
3. the 2L partial message candidate $\hat{M}$ on each restart location R(ρ)∈R.

As a consequence, the memory overhead depends on *R* but also on the positions of the restart locations, since the partial message is stored. For the ρ-th restart location, $2\times L\times R_{A}\left(\rho \right)$ bits are required to store the partial messages. Moreover, the information on the 2L path is stored, as discussed in Section 3.3. The total memory overhead of the LLRM is denoted as $\Lambda_{\mathrm{LLRM}}$ and is

(23) $$\Lambda_{\mathrm{LLRM}}=\underset{R}{\underset{︸}{n\cdot R}}+\underset{P}{\underset{︸}{R\cdot 2L\cdot Q_{\mathrm{PM}}}}+\underset{\hat{M}}{\underset{︸}{\sum_{\rho =1}^{R}2LR_{A}\left(\rho \right)}}.$$

**Figure 5:**
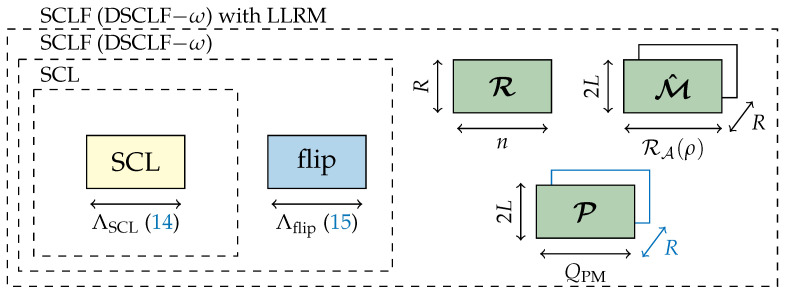
Memory sketch of SCLF with the LLRM.

The memory sketch of the SCLF decoder with the LLRM is provided in Figure 5. The green blocks correspond to the memory overhead (23) when embedding the proposed LLRM.

## 4. Obtaining the Restart Locations

In this section, three designs for the set of restart locations are proposed. The choice of restart locations aims at improving the execution time reduction provided by the LLRM. Two are based on the structure of the polar code, while one is proposed based on offline simulations. For the latter, similarly to [17], offline simulations are used to retrieve the probability-mass function (PMF) of the first path-flipping candidate $\min(\varepsilon_{t})$ for list–flip decoders. These statistics are then used to design the set R.

### 4.1. Structural Design

Before describing the simulation-based design, two simple sets of restart locations are described. Both sets use the structure of the polar code. The first design of restart locations, noted as $R_{\mathrm{divN}}$, equally divides the codeword into *R* segments. Since the codeword is of length *N*, each segment is of size $\frac{N}{R}$. This particular design does not take into account the information set and is constructed as $R_{\mathrm{divN}}=\left\{0,N/R,\ldots ,\frac{N(R-1)}{R}\right\}$. For R=2, the restart locations $R_{\mathrm{divN}}=\left\{0,\frac{N}{2}\right\}$ correspond to the restart locations of the SRM [16]. Restarting at 0 does not allow us to save any computations, which limits the effect of this mechanism for high-rate codes, as discussed in [16].

The second design, generating $R_{\mathrm{divK}}$, defines the restart locations on the basis of the information set A. With the exception of the first restart location, any two successive restart locations are separated by $\left\lceil \frac{K+r}{R}\right\rceil$ information bits. The first restart location is $R_{\mathrm{divK}}\left(1\right)=A_{\log_{2}\left(L\right)+1}$, since no restart is possible if $a\notin A_{\mathrm{sort}}$. All restart locations correspond to $R_{\mathrm{divK}}=\left\{a_{\log_{2}\left(L\right)+1},a_{\left\lceil (K+r)/R\right\rceil },\ldots ,a_{\left\lceil \frac{\left(K+r)(R-1\right)}{R}\right\rceil }\right\}$ or $R_{A}=\left\{\log_{2}\left(L\right)+1,\left\lceil \frac{K+r}{R}\right\rceil ,\ldots ,\left\lceil \frac{\left(K+r)(R-1\right)}{R}\right\rceil \right\}$. For any additional trials, part of the computations will be avoided since $R_{\mathrm{divK}}\left(1\right)\neq 0$. This design pushes the restart locations towards the end of the codeword, allowing us to avoid many computations. However, these restart locations may not often used since $\min(\varepsilon_{t})$ is expected to be close to the first information-bit indices.

### 4.2. Design Based on the First Path-Flipping Location

In the following, the value of the first path-flipping location is denoted as $i_{1}=\min\left(\varepsilon_{t}\right)$. The simulation-based design requires us to know the probability that $i_{1}$ is the first path-flipping location. Next, the probability that $a\in A_{\mathrm{sort}}$ is the first path-flipping location occurring during an additional trial of list–flip decoders is denoted by $P(i_{1}=a)$. The algorithm to obtain the PMF by simulation is described in Algorithm 1. A design signal-to-noise ratio (SNR) is chosen to match a desired FER. Moreover, the list size *L* and the number of additional trials $T_{\max}$ are chosen to define the list–flip decoder. If the decoder performs an additional trial, the value of $i_{1}=\min\left(\varepsilon_{t}\right)\in A_{\mathrm{sort}}$ is stored in a structure denoted as Occ. After transmitting *C* codewords, with *C* being large enough, a reliable probability distribution describing $P(i_{1}=a)$ for all $a\in A_{\mathrm{sort}}$ calculations is returned. This distribution is then used to choose the restart location R.
**Algorithm 1** Obtaining the distribution of the first path-flipping candidates occurring in SCLF by simulation1: **procedure** Distr_Bit_Flip_SCLF($C,\;A,\;T_{\max},\;L,\;\mathrm{SNR}$)
2:     **for** $j=\log_{2}\left(L\right)+1:\left|A\right|$ **do**

3:         $P\left(i_{1}=a_{j}\right)\leftarrow 0$▹ Initialize $P,\forall a\in A_{\mathrm{sort}}$4:         $\mathrm{Occ}\left(j\right)\leftarrow 0$▹ Initialize counter $\forall a\in A_{\mathrm{sort}}$5:     **end for**
6:     $T_{\mathrm{sim}}\leftarrow 0$▹ Total number of additional trials performed
7:     **for** c=0:C−1 **do**
8:         x←
Polar_Encoding(u)

9:         $\alpha_{\mathrm{ch}}\leftarrow \mathrm{AWGN}\left(\mathrm{SNR},x\right)$▹ Channel LLRs for the decoding10:         $\left(B_{\mathrm{flip}},t\right)\leftarrow \mathrm{SCLF}\left(\alpha_{\mathrm{ch}},A,T_{\max}\right)$▹ $B_{\mathrm{flip}}$: Set of flipping locations in SCLF, *t*: Number of additional trials performed11:         **if** t>0 **then**▹ Initial trial has failed12:            $T_{\mathrm{sim}}\leftarrow T_{\mathrm{sim}}+t$▹ Update the total number of additional trials13:            **for** τ=1:t **do**
14:                $\varepsilon_{\tau }\leftarrow B_{\mathrm{flip}}\left(\tau \right)$
15:                $a_{k}\leftarrow \min\left(\varepsilon_{\tau }\right)$▹ Extract first bit-flipping $a_{k}\in A_{\mathrm{sort}}$16:                $\mathrm{Occ}\left(k\right)\leftarrow \mathrm{Occ}\left(k\right)+1$▹ Increase by 1 the occurence $i_{1}=a_{k}$17:            **end for**18:         **end if**
19:     **end for**20:     **for** $j=\log_{2}\left(L\right)+1:\left|A\right|$ **do**
21:         $P\left(i_{1}=a_{j}\right)\leftarrow \mathrm{Occ}\left(j\right)/T_{\mathrm{sim}}$▹ Estimate the probability-mass function $P(i_{1}=a_{j})$22:     **end for**
23:     **return** the distribution $P\left(i_{1}=a\right),\forall a\in A_{\mathrm{sort}}$
24: **end procedure**


Ultimately, the design consists of choosing the restart locations by equally dividing the distribution in segments sharing the same probability of having $\min(\varepsilon_{t})$. The algorithm to obtain the set of restart locations $R_{\mathrm{prob}}$ according to the PMF is described in Algorithm 2. The number of restart locations $R=\left|R\right|$ is selected in advance. The restart location R(ρ), 1≤ρ≤R, is the first location verifying $P\left(i_{1}=R\left(\rho \right)\right)>\frac{\left(\rho -1\right)}{R}$. We note that $R\left(1\right)$ is set to the smallest $a\in A_{\mathrm{sort}}$, verifying $P(i_{1}=a)>0$, and it is usually $a_{\log_{2}\left(L\right)+1}$.
**Algorithm 2** Design of R with the distribution of the first path-flipping location1: **procedure** Design_Restart_with_PMF(P,R,A)2:     sumP←03:     ρ←14:     **for** $j=\log_{2}\left(L\right)+1:K+r$ **do**
5:         $sumP\leftarrow sumP+P\left(i_{1}=a_{j}\right)$6:         **if** $sumP>\frac{\rho -1}{R}$ **then**▹ Divide equally with resp. to P7:            $R\left(\rho \right)\leftarrow a_{j}$8:            ρ←ρ+1▹ Next restart location9:         **end if**10:         **if** ρ>R **then**11:            **return** R▹ Already *R* restart locations in R12:         **end if**13:     **end for**14: **end procedure**

### 4.3. Simulation Setup and Results

For this analysis, a polar code of length N=1024 with rate ½ and a CRC of r=16 bits is simulated over the AWGN channel with the BPSK modulation. Simulations are for a minimum of $C=10^{5}$ codewords and are run until at least $10^{3}$ errors are observed. The target FER is $10^{-2}$ as in [17], which is obtained at $E_{b}\;/\;N_{0}=1.625$ dB. The DSCLF-ω decoder with ω=3 and $T_{\max}=300$ is simulated. The list size is set to L=2. The value of $T_{\max}$ was selected to achieve the optimal error-correction performance at the target FER.

**Figure 6:**
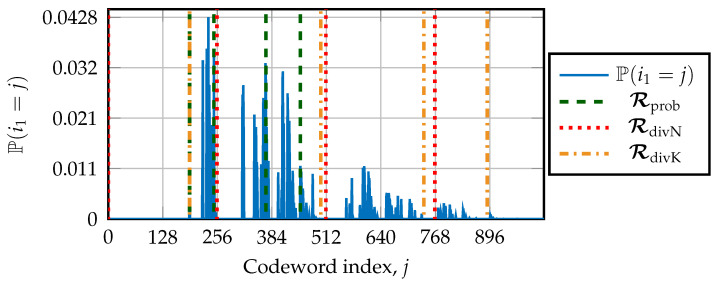
Distribution describing $P(i_{1}=j)$ under DSCLF-3 decoding for (1024,512+16) code. Vertical lines indicate restart locations R.

The PMF is depicted in Figure 6. In the considered example, the set of restart locations is R=4. Locations found by Algorithm 2 are denoted by $R_{\mathrm{prob}}$ and equally divide the distributions according to their probabilities. For this example, the restart locations are $R_{\mathrm{prob}}=\left\{191,248,370,451\right\}$. Regarding the structural designs, the restart locations are $R_{\mathrm{divN}}=\left\{0,256,512,756\right\}$ and $R_{\mathrm{divK}}=\left\{191,499,741,890\right\}$.

## 5. Simulation Results

Next, simulation results are provided. This section comprises error-correction performance, memory estimations, and average execution time reductions for list–flip decoders. The polar codes are constructed with a design SNR $E_{b}\;/\;N_{0}=\left\{1.5,2.0,3.4\right\}$ dB for rates $R_{code}$ = {¼, ½, ¾}, respectively. For all simulations, the number of restart locations is set to R=4. The list size is selected as L=2 to maintain a low-complexity list decoder. The maximum number of trials is selected as $T_{\max}=\left\{30,50,300\right\}$ for SCLF and DSCLF-ω decoders with ω={2,3}, respectively. The BPSK modulation is used over an AWGN channel.

### 5.1. Error-Correction Performance

**Figure 7:**
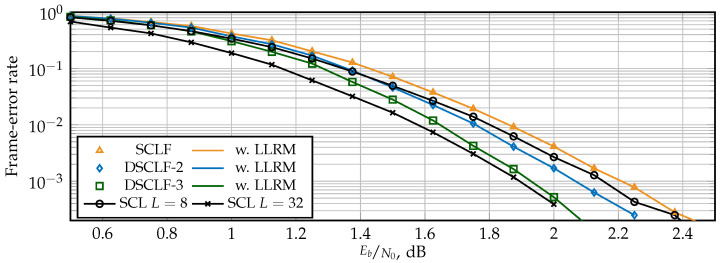
Error-correction performance of SCLF and DSCLF decoders with L=2 for R= ½ codes and N=1024.

**Figure 8:**
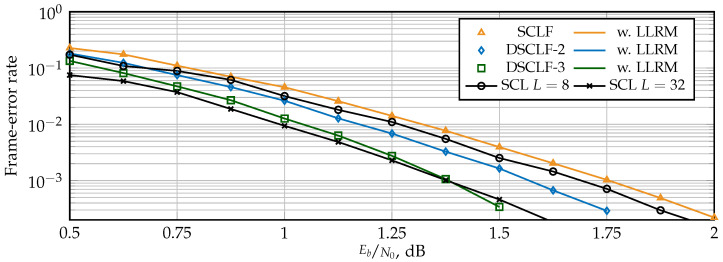
Error-correction performance of SCLF and DSCLF decoders with L=2 for R= ¼ codes and N=1024.

The FER for SCLF and DSCLF-ω with various ω are shown in Figure 7 for polar codes of N=1024 with rate $R_{code}$ = ½ and in Figure 8 for polar codes with rate $R_{code}$ = ¼. For reference, standard SCL decoders with L=8 and L=32 are provided. The LLRM is derived from the GRM, a mechanism that does not affect error-correction performance [17]. Both figures show that the proposed LLRM does not affect the error-correction performance of the original SCLF and DSCLF decoders either. Moreover, higher order DSCLF decoders greatly increase the error-correction performance compared to SCLF. The performance of DSCLF-3 with $T_{\max}=300$ is close to the performance of the SCL decoder with L=32 for both rates and even matches for $R_{code}$ = ¼ and $\mathrm{FER}=10^{-3}$.

### 5.2. Memory Estimations

Memory requirements are estimated with (14), (15), (19), and (23), where the same quantization scheme as that of [16,23] is used. Hence, the blocks based on LLR values are quantized by $Q_{\mathrm{ch}}=6$, $Q_{\mathrm{int}}=7$, $Q_{\mathrm{flip}}=7$ bits, and $Q_{\mathrm{pm}}=8$ bits, respectively. The sizes of these blocks also depend on the values of *N*, *L*, and $T_{\max}$. The number of restart locations R=4 is selected for all estimations, which was similarly carried out in the example in Section 4. All sets of restart locations are considered. Table 1 provides the memory overhead, expressed in percent, induced by embedding the restart mechanism to the polar code decoder for code lengths N={512,1024,2048} and rates $R_{code}$ = {¼, ½, ¾}. For N=1024, the memory overhead is given for SCLF, DSCLF-2, and DSCLF-3, while for N={512,2048}, the memory overhead for DSCLF-3 is provided. By analyzing Table 1, the memory overhead to embed the LLRM depends on the set of restart locations R, since the location affects the size of $\hat{M}$ in (23). Moreover, for $R_{\mathrm{divK}}$, the increase in the code rate increases the memory overhead since the size of $\hat{M}$ becomes larger. For a code rate of $R_{code}=$ ¼, the memory overhead is around 1.5% for $R_{\mathrm{prob}}$ while it is 5% for $R_{\mathrm{divK}}$. Meanwhile, for the GRM with $R_{\mathrm{GRM}}=A_{\mathrm{sort}}$ ($|A_{\mathrm{sort}}|=K+r-\log_{2}\left(L\right)$), the memory overhead depends on the code rate and the code length but not the decoder. By doubling the code length *N*, the overhead approximately doubles as well. For N=512 and rate $R_{code}=$ ¾, the overhead is 623.8% and 3204.5% for N=2048. Hence, Table 1 shows that the overhead induced by the GRM makes it unfeasible. However, the overhead induced by the LLRM remains feasible for an implementation.

### 5.3. Average Execution Time Reduction Induced by the LLRM

Next, with respect to the standard list–flip decoder (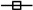), the reduction brought by the LLRM is estimated for various code lengths, code rates, and various restart locations designed according to simulations $R_{\mathrm{prob}}$ (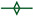) or designed according to code properties such as $R_{\mathrm{divN}}$ (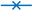) and $R_{\mathrm{divK}}$ (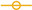). In order to compute it, the chosen architectural execution-time model is from [17]. Moreover, the number of processing elements, having an impact on the average execution time of decoders, is P=64 as in [23,25]. Moreover, the latency of SCL is estimated as in [15], i.e., one clock cycle is added whenever the SCL encounters an information bit.

Two types of reduction will be discussed next, the average execution time reduction ($\Delta_{\mathrm{GRM}}$ and $\Delta_{\mathrm{LLRM}}$) and the average flip time reduction ($\Delta_{\mathrm{GRM}}^{\mathrm{flip}}$ and $\Delta_{\mathrm{LLRM}}^{\mathrm{flip}}$). The first consists of the reduction over all the simulated frames. The second consists of the reduction over the time spent during the flip part of the list–flip algorithm; since the flipping part is not required in all frames, this reduction is expected to be greater, i.e., $\Delta_{\mathrm{LLRM}}^{\mathrm{flip}}>\Delta_{\mathrm{LLRM}}$ ($\Delta_{\mathrm{GRM}}^{\mathrm{flip}}>\Delta_{\mathrm{GRM}}$). The reduction brought by the unfeasible GRM serves as a bound, i.e., $\Delta_{\mathrm{GRM}}>\Delta_{\mathrm{LLRM}}$ and $\Delta_{\mathrm{GRM}}^{\mathrm{flip}}>\Delta_{\mathrm{LLRM}}^{\mathrm{flip}}$, and the GRM reduction is shown in the figures as (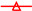).

**Figure 9:**
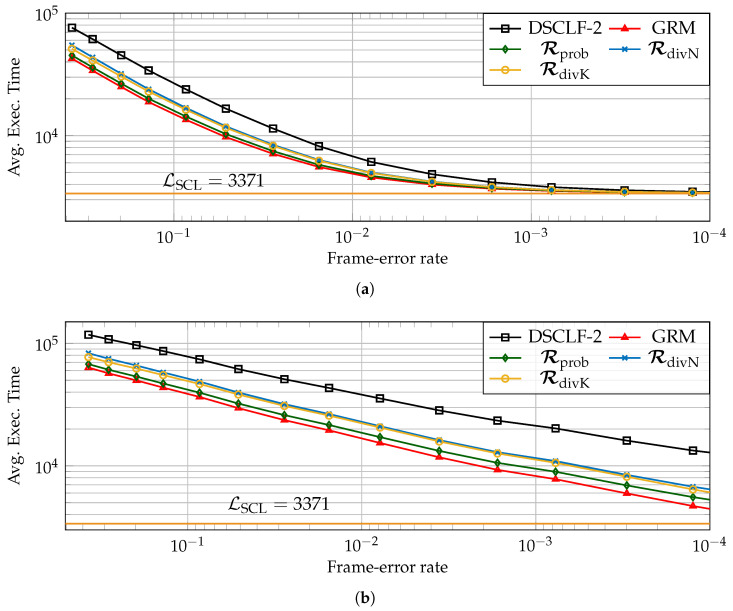
Execution time (**a**) and execution flip time (**b**) of DSCLF-2 decoder of polar codes with rate ¼ and N=1024.

**Figure 10:**
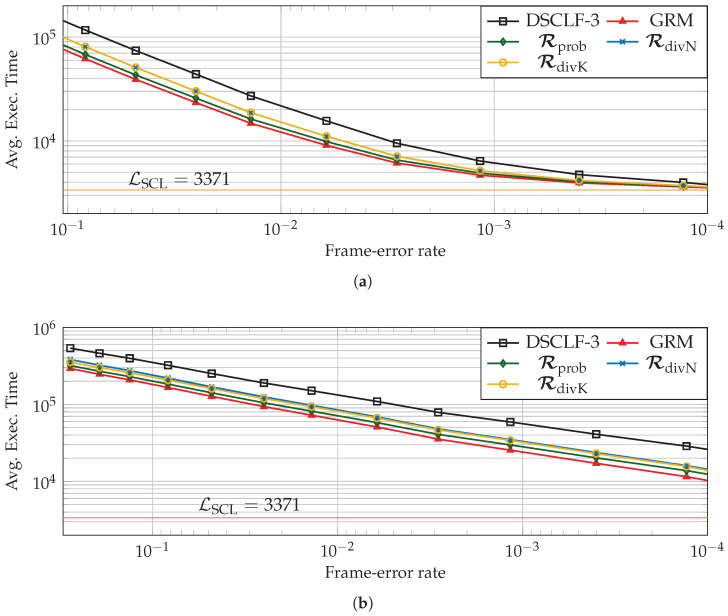
Execution time (**a**) and execution flip time (**b**) of DSCLF-3 decoders of polar codes with rate ¼ and N=1024.

Figure 9 and Figure 10 depict the average execution time over all frames (a) and over the flipping part (b) for the DSCLF-2 and DSCLF-3 decoders for the (1024,256+16) polar code. For both decoders, the reduction is clearly visible for all sets of restart locations. However, the set of restart locations $R_{\mathrm{prob}}$ allows for a greater reduction and is closer to the optimal reduction retrieved with the GRM. The reduction over all frames reduces with the FER since the flipping part is required less often, and the mechanism permits a reduction only on the flipping part. In Figure 10a, the reduction with $R_{\mathrm{prob}}$ at FER=0.1 is $\Delta_{\mathrm{LLRM}}=41.8$% while the reduction is $\Delta_{\mathrm{LLRM}}=4.7$% at $\mathrm{FER}=10^{-4}$. If the focus is made on the flipping part solely, the reduction in the average flip time increases when the FER diminishes. In Figure 10b, the reduction with $R_{\mathrm{prob}}$ at FER=0.1 is $\Delta_{\mathrm{LLRM}}^{\mathrm{flip}}=43.0$%, while the reduction is $\Delta_{\mathrm{LLRM}}^{\mathrm{flip}}=52.9$% at $\mathrm{FER}=10^{-4}$.

Table 2 recapitulates all aforementioned reductions with respect to the standard decoding for N={512,1024,2048} and code rates K/N= {¼, ½, ¾}. For N=1024, the execution time reduction by embedding one of the restart mechanisms on SCLF and DSCLF-2 is also given. The reduction is computed at $\mathrm{FER}=10^{-2}$ as in [17]. Regardless of the decoders, code lengths, or code rates, the proposed design $R_{\mathrm{prob}}$ provides the best reduction. The reduction induced by the LLRM with $R_{\mathrm{prob}}$ is highlighted in boldface. As the code rate increases, the reduction reduces since the restart locations tend towards the beginning of the decoding tree, as explained in [16,17]. A reduction of 51.7% with respect to the DSCLF-2 algorithm is estimated for the code rate ¼ and N=1024. For these parameters, the other designs provide a reduction of 40.7 and 42.1%. The smallest reduction observed is for DSCLF-2 with rate ¾ and N=1024. The reduction is 7.0%, while the optimal reduction is 9.8%. On average, 3 to 7% is lost with respect to the reduction provided by the GRM.

## 6. Conclusions

In this paper, a restart mechanism is proposed for list–flip decoders of polar codes. We first show that an optimal mechanism is unfeasible due to a large memory overhead, which can increase up to 3755%. Thus, an limited-locations restart mechanism (LLRM) is proposed, allowing us to restart in predefined locations of the tree if an additional trial is performed. This mechanism requires the storage of path information at these locations. The choice of the restart locations influences the effectiveness of the LLRM. Three designs of restart locations are proposed and compared to each other. A thorough analysis is performed for various list–flip decoders, as well as various code lengths and code rates. The design requiring simulations achieves a reduction of 41.7% with respect to the DSCLF-3 decoding of the (1024,256+16) code at the cost of 1.5% memory overhead. This reduction is only 4% smaller than the optimal mechanism which comes at the cost of 177% memory overhead.

## Figures and Tables

**Table 1:** Memory estimates and overhead for SCLF-based decoders with GRMs, LLRMs, and the original decoders.

*N*	ω	$T_{\max}$	$\Lambda_{\mathrm{list}\text{-}\mathrm{flip}}$	$R_{\mathrm{code}}$	$\Delta_{\mathrm{mem}}^{\mathrm{LLRM}}$			$\Delta_{\mathrm{mem}}^{\mathrm{GRM}}$
					$R_{\mathrm{prob}}$	$R_{\mathrm{divK}}$	$R_{\mathrm{divN}}$	
			bits		%	%	%	%
1024	1	30	32,270	¼	1.8	5.5	2.1	235.0
				½	7.7	10.3	6.1	875.1
				¾	4.6	15.0	12.0	1921.5
1024	2	50	33,110	¼	2.2	5.5	2.1	229.0
				½	2.2	10.0	6.0	853.0
				¾	2.7	14.7	11.7	1872.7
1024	3	300	42,860	¼	1.5	4.2	1.6	176.9
				½	1.4	7.7	4.6	658.9
				¾	1.8	11.3	11.7	1446.7
512	3	300	21,682	¼	1.7	3.9	1.7	83.1
				½	2.4	6.8	4.2	290.7
				¾	1.8	9.8	7.6	623.8
2048	3	300	75,504	¼	1.5	4.4	1.6	374.0
				½	1.6	8.5	4.9	1442.1
				¾	1.1	12.5	13.0	3204.5

**Table 2:** Execution time reduction by embedding the LLRM and the GRM to list–flip decoders of various polar codes at the FER of $10^{-2}$.

*N*	ω	$T_{\max}$	$R_{\mathrm{code}}$	$E_{b}\;/\;N_{0}$	$\Delta_{\mathrm{GRM}}$	$\Delta_{\mathrm{LLRM}}$ (%)			$\Delta_{\mathrm{GRM}}^{\mathrm{flip}}$	$\Delta_{\mathrm{LLRM}}^{\mathrm{flip}}$ (%)		
				dB	(%)	$R_{\mathrm{prob}}$	$R_{\mathrm{divN}}$	$R_{\mathrm{divK}}$	(%)	$R_{\mathrm{prob}}$	$R_{\mathrm{divN}}$	$R_{\mathrm{divK}}$
1024	1	30	¼	1.34	18.5	16.9	12.3	14.3	51.9	47.4	34.6	40.1
			½	1.87	12.8	9.9	7.9	8.1	35.8	27.7	22.1	22.7
			¾	3.03	10.4	7.8	7.1	5.9	28.3	21.0	19.1	16.1
1024	2	50	¼	1.21	25.4	23.1	18.2	18.9	56.7	51.7	40.7	42.1
			½	1.78	19.1	14.8	12.5	12.2	36.0	27.9	23.5	22.9
			¾	3.00	9.8	7.0	5.8	5.1	23.1	16.5	13.7	12.0
1024	3	300	¼	1.06	45.5	41.7	31.0	33.2	52.0	47.6	35.4	37.9
			½	1.66	27.8	22.2	16.7	17.2	32.2	25.7	19.3	19.9
			¾	2.86	15.3	11.7	7.1	7.5	18.7	14.3	8.7	9.2
512	3	300	¼	1.39	47.3	43.3	34.4	35.5	55.7	51.0	40.5	41.9
			½	1.92	30.8	28.1	20.0	20.3	35.5	32.4	23.1	23.4
			¾	3.08	14.5	11.5	6.6	6.8	18.2	14.4	8.2	8.5
2048	3	300	¼	0.84	44.1	38.0	31.8	26.7	55.2	47.6	39.8	33.4
			½	1.49	27.8	21.8	17.2	17.7	33.7	26.5	20.8	21.5
			¾	2.73	12.5	8.9	4.7	4.8	15.5	11.0	5.8	6.0

## Data Availability

The original contributions presented in the study are included in the article; further inquiries can be directed to the corresponding authors.

## Abbreviations

The following abbreviations are used in this manuscript:

AWGN	additive white Gaussian noise
BPS	binary phase-shift keying
CA	CRC-aided
CRC	cyclic redundancy check
DSCF	dynamic successive-cancellation flip
DSCLF	dynamic successive-cancellation list flip
eMBB	enhanced mobile broadband
FER	frame-error rate
GRM	generalized restart mechanism
LLR	log-likelihood ratio
LLRM	limited location restart mechanism
PM	path metric
PMF	probability-mass function
PS	partial sum
PSCLF	successive-cancellation list flip
RHS	right-hand side
SC	successive cancellation
SCL	successive-cancellation list
SCLF	successive-cancellation list flip
SCF	successive-cancellation flip
SCF	simplified restart mechanism
SNR	signal-to-noise ratio
